# Cyber-Attack Prediction Based on Network Intrusion Detection Systems for Alert Correlation Techniques: A Survey

**DOI:** 10.3390/s22041494

**Published:** 2022-02-15

**Authors:** Hashim Albasheer, Maheyzah Md Siraj, Azath Mubarakali, Omer Elsier Tayfour, Sayeed Salih, Mosab Hamdan, Suleman Khan, Anazida Zainal, Sameer Kamarudeen

**Affiliations:** 1 School of Computing, Faculty of Engineering, Universiti Teknologi Malaysia (UTM), Skudai Johor 81310, Malaysia; hthashim2@graduate.utm.my (H.A.); anazida@utm.my (A.Z.); 2 College of Computer Science, King Khalid University, Abha 61421, Saudi Arabia; aabdurrahman@kku.edu.sa (A.M.); oalser@kku.edu.sa (O.E.T.); sameer@kku.edu.sa (S.K.); 3 College of Computer and Information Sciences, King Saud University, Riyadh 11461, Saudi Arabia; salih.sayd@gmail.com; 4 Department of Computer Science, University of São Paulo, São Paulo 13566-590, Brazil; mosab.hamdan@ieee.org; 5 School of Psychology and Computer Science, University of Central Lancashire, Preston PR1 2HE, UK; skhan92@uclan.ac.uk

**Keywords:** intrusion detection, alerts correlation, attacks prediction, machine learning

## Abstract

Network Intrusion Detection Systems (NIDS) are designed to safeguard the security needs of enterprise networks against cyber-attacks. However, NIDS networks suffer from several limitations, such as generating a high volume of low-quality alerts. Moreover, 99% of the alerts produced by NIDSs are false positives. As well, the prediction of future actions of an attacker is one of the most important goals here. The study has reviewed the state-of-the-art cyber-attack prediction based on NIDS Intrusion Alert, its models, and limitations. The taxonomy of intrusion alert correlation (AC) is introduced, which includes similarity-based, statistical-based, knowledge-based, and hybrid-based approaches. Moreover, the classification of alert correlation components was also introduced. Alert Correlation Datasets and future research directions are highlighted. The AC receives raw alerts to identify the association between different alerts, linking each alert to its related contextual information and predicting a forthcoming alert/attack. It provides a timely, concise, and high-level view of the network security situation. This review can serve as a benchmark for researchers and industries for Network Intrusion Detection Systems’ future progress and development.

## 1. Introduction

Network Intrusion Detection Systems (NIDs) are rapidly becoming ubiquitous due to sophisticated risk associated with network attacks. The Network Intrusion Detection Systems (NIDSs) are designed to safeguard the security needs of enterprise networks against cyber-attacks. NIDS networks suffer from several limitations, such as the generation of a high volume of low-quality alerts. Moreover, 99% of the alerts, produced by NIDSs, are false positives [1,2]. The Alert Correlation (AC), which is known as IDS post-processing, has been proposed to overcome these limitations. The Intrusion Alert Prediction can assist early caution and prevention to avoid the attacks from escalating and damaging the network as a proactive approach [3].

An intrusion can be characterized as a framework transgression of the security policy that refers to the security components, which are committed to identify infringement of framework security. Intrusive activities are not the same as from normal system activities and the abnormal system activities. Intrusion Detection Systems (IDSs) do not replace other security strategies, for example, verification and access control preventions methods, as they are integral for the existing security. Distinctive advances approaches are utilized as a part of IDS, including data mining [4], machine learning [5], hidden Markov models [6], honeypot [7], genetic algorithms [8], and fuzzy [9], deep learning [10]. In addition, different types of IDSs have additionally been created, which incorporate the Network Intrusion Detection Systems (NIDS), Host-Based IDS, Stack-Based IDS, Protocol-Based IDS (PIDS), and Graph-Based IDS [11,12]. The technique, like packet sniffing, is used by NIDS, which helps in examining the collected network information. In addition, it tries to find access to a computer network that is unauthorized. A commonplace NIDS incorporates various sensors to observe packet traffic, administration capacities, and at least one administration reassures for human interface.

Table 1 shows the comparison of proposed survey with the existing survey articles, such as [13,14,15,16] focus on alert correlation techniques or a mapping among framework techniques and components. No articles comprehensively reviewed cyber-attack prediction based on intrusion alert correlation techniques, considering the intrusion alert dataset. In addition, the development of alert correlation systems has been such that several different systems have been proposed in the meantime, and so there is a need for an update. The taxonomy of alert correlation approaches and components is presented in this paper. In view of the discussion on prior surveys, this article focuses on the following:

- State of the art intrusion alert prediction methods.
- Presenting a classification and comparison of alert correlation approaches.
- State of the art intrusion alert datasets, which are not considered in the existing surveys.

The survey is organized as follows: The state-of-the-art intrusion alert prediction models are presented in Section 2. In Section 3, we present an alert correlation taxonomy. A generalized component in intrusion alert correlation models are presented in Section 4. In Section 5, presented a number of datasets have been used for testing Alert Correlation research. Some discussion and future research direction are reviewed in Section 6. Finally, we conclude the survey in Section 7.

## 2. State-of-the-Art Intrusion Alert Prediction Models

NIDS technology plays an essential role in protecting communication networks from cybercrime. However, these technologies are not viable in predicting future attacks. It produces much of alerts when attack activities/intrusions have effectively occurred. A proactive approach is to predict and lead conceivable attacks to prevent damage. However, network intrusion prediction is still an active investigation.

This section presents current methodologies for prediction algorithms, which considered as applicable to predicting intrusion alerts. Their main advantages and disadvantages are summarized in Table 2.

### 2.1. Plan Library

The plan is utilized for predicting an attacker’s behavior [17,18]. defined how a plan library of specific attacks to predict an attack plan. The security expert is required to accumulate the plan library manually. However, this can be time-consuming, and it is not always able to reply to a new form of attack variants. The complexity of plan matching increases because of variation of missing actions in an attack sequence. Therefore, the plan library expected to be updated regularly to ensure it meets the new attack sequence.

### 2.2. Network Attack Graph

The process of utilization of a network attack in the form of a graph is undertaken to discover the available security vulnerabilities and find all possible attack sequences [19]. The network attack graph is made of correlating intrusion alerts that are in accordance with source and destination Internet Protocol (IP) addresses. The anticipated next alert is determined through predictability scores resulting after the attack graph. It provides the graphical stream relating to the attack sequence to the network administrator. In order to enhance the prediction, some alerts need to be removed manually because the generated graph includes low probable alerts in attack sequence. However, the method cannot discover out-of-sequence attacks.

### 2.3. Sequence Pattern Mining

The technique, known as sequence pattern mining, decreases the exertion to advance pattern rules [20]. A historic attack sequence considered to be vulnerable several recent attack strategies, while making sure it utilizes the resultant database. The authors of [21] observed that many of the attacks are normally completed within a specific time span and put forward in an incremental mining algorithm to differentiate sequential attack patterns over the separated time window. The database is rationalized within a smaller period afterward the arrival of a new form of attack procedure. After the fundamental regulation generation, the performance of subsequent updates is speedier as the amount of new alert sequence then decreases.

### 2.4. Machine Learning and Data Mining

The machine learning procedures are utilized to learn past behavior of the attackers. Afterward, the knowledge is utilized to anticipate the subsequent stages of a future attack in real time [22,23]. The authors of [24] presented a prediction algorithm, known as Nexat. It comprises three operational stages, which incorporate the information extraction stage, the preparation stage, and the expectation stage. At run time, it utilizes the trained database and weighted probability to anticipate the next alert. The historical database that is large enough is required to find the next fit to the historical data; it cannot predict the new attacks.

### 2.5. Relational Time Series

Another way to process intrusion alerts is carried out by arranging them in relational time series (RTS) form [25]. The IDS generates certain security alerts in sequential order by time of arrival. The relational percepts are formed by these alerts sequence. Every percept is characterized as $pi=r(c_{1},c_{2}$…$c_{m}$), where r represents the predicate and $c_{i\in \left(1\ldots m\right)}\;$are constants that denote objects. For security alerts, r is the ready type/identity and $c_{i\in \left(1\ldots m\right)}$ refers to an entity, for example, source or destination IP. The main aim of the technique is to predict the steps of the future attacker, which can be observed as future NIDS alerts.

### 2.6. Bayesian Network

The Bayesian network-based approach has been proposed for learning an attack strategy to correlate alert and predict conceivable forthcoming attacks in an online system [26,27]. The general architecture of this intrusion alert prediction model includes two segments. The offline attack pattern recognition is utilized to remove attack action patterns automatically, which create correlation rules by examining alert using Bayesian networks. The online alert correlation is associated with alerts in three steps that include alert fusion, attack thread reconstruction, and attack scenario reconstruction.

### 2.7. Deep Learning

Recently, Deep Learning techniques have made great progress in many domains, not limited to the field of network security (NIDS). These techniques/approaches aim to observe/encode/predict/classify patterns or pattern sequences by learning a good feature representation. Some of the most successful deep learning methods involve artificial neural networks, such as stacked auto-encoder (SAE), convolutional neural networks (CNN), recurrent neural networks (RNN), deep belief networks (DBN), and reinforcement learning.

Studies show that deep learning completely betters than traditional methods in most of areas. The most important advantage of deep learning is replacing handcrafted features with efficient algorithms for unsupervised or semi-supervised feature learning and hierarchical feature extraction [28,29].

Works in [3,30,31] proposed a deep learning method for intrusion alert prediction.

## 3. Taxonomy

**Figure 1:**
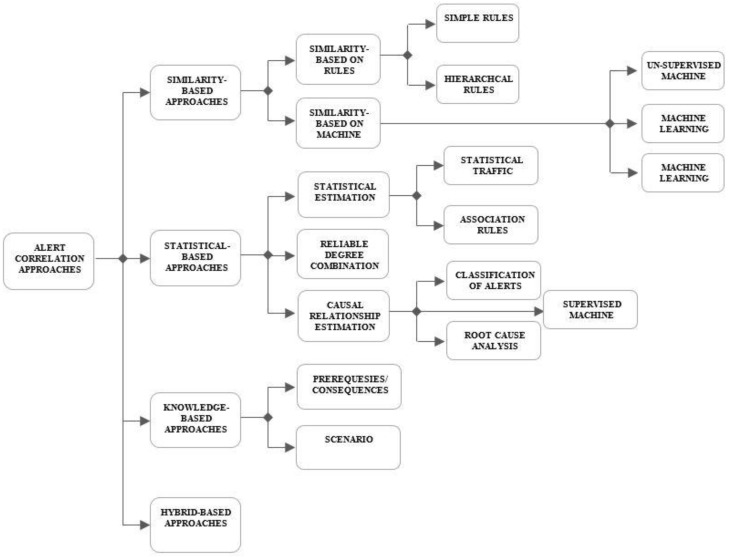
Taxonomy of alert correlation approaches.

NIDS post-processing has been studied for over 10 years to overcome the limitation of NIDS, particularly a high volume of low-quality alerts. In Figure 1, we present a taxonomy of existing alert correlation approaches, which can be categorized as similarity-based [33], statistical-based [34], and knowledge-based alert correlation [16,35]. Regardless of these approaches, a hybrid-based approach has also been shown [36,37]. The similarity-based approach focusses on addressing the issue of enhancing the nature of alerts attributes. The statistical-based approaches are managing the issue of perceiving the attack scenario, in view of the statistical or causal relationship between alerts; knowledge-based approaches are dealing with attack definition considering ready importance. Additionally, a hybrid-based approach can exploit the qualities of three correlation approaches.

### 3.1. Similarity-Based Approach

The similarity-based approach is defined as a measure to find the similarity between two alerts or alert clusters. This approach clusters similar alerts in time to reduce the number of alerts and increase its ability to discover the known attacks. The approach can discriminate between false positive, redundant, and invalid alerts, whereas the types of attacks do not need to be defined. The similarity between alerts can be modeled based on simple rules, hierarchal rules, and automatic generation using machine learning:

1. Similarity-based on Simple Rules—The Simple Rule similarity-based algorithm defines a simple rule to measure the similarity between alert attributes, which can be aggregated together [38,39,40].
2. Similarity-based on Hierarchal Rules—The Hierarchal Rule similarity-based algorithm defined similarity measures in a generalized hierarchical concept. It has been defined as a similarity that works on the basis of root cause analysis to detect root causes in networks [41,42]. The root of cause of similar alerts are same to the root cause of hierarchy.
3. Similarity-based on Machine Learning—The similarity measures are generated automatically. The pre-requisite for supervised algorithms characterizes the existence of clustered alerts, which is set by the machine-learning algorithms. Algorithms, without this requirement (unsupervised), require training to measure the similarity between alerts. A neural network used to perform clustering based on alert reconstruction error [5,27,43,44]. It is a one-step process of clustering and decision-making based on cluster statistics. The online learning capability applies false and true alert patterns on the basis of labeled data.

### 3.2. Knowledge-Based Approach

Existing works of this approaches are based on the knowledgebase of attack definitions, which has been divided into two components:

1. Scenario—The principle utilization of scenario-based or pre-defined scenario approaches is to predict the multi-step attacks, as a real attack consists of a sequence of steps [27,45]. An attack scenario is defined by its relating steps or stages, which are required to succeed. Different attack scenario modeling languages are presented, such as [19,27,45,46,47], However the main idea in all of them is to define the stages and preconditions of an attack, as well as its goals based on the rules stored in the knowledge base, which specifies that the stages of the attack are necessary to achieve success. The knowledge rules are constructs either by machine learning or manually by experts. However, the correlation rules in machine learning approaches are obtained using the training stage and labeled data.
2. *Prerequisites/Consequences*—These methodologies observe and control implications of alerts and existing knowledge in the network and then detect/predict the security event. If some post-conditions of the first alert match some pre-conditions of the next alert, then the two alerts can be logically correlated. However, a complete scenario description is not required here.
  One of the first algorithms to use background knowledge has been proposed by [48]. They used provides/requires a model for describing causal relationships between alerts using JIGSAW language. However, it can only detect known attacks. Attack scenarios are modeled in terms of concept and capabilities. Concepts are an abstraction of attacks, and functionality is a mandatory and provided condition associated with each attack concept. The correlation task runs when a match is found between the conditions of two temporally ordered alerts. Therefore, each alert received is modeled into a concept with the relevant required and provided capabilities. It does not represent the attack scenario as a set of states, but as a set of concepts and capabilities.

Several efforts based on this model have been proposed, and they use different definitions and epistemological representations [22,49,50].

### 3.3. Statistical-Based Approach

The basic idea of these approaches is that relevant attacks have similar statistical features, and a proper classification can be found by detecting these similarities. These approaches do not need any context knowledge. They store the causal relationship between different events and use previous statistical analysis of data to analyze the frequency of occurrence during system training, and then generate attack steps. This knowledge is used for correlating different attack steps after learning these relationships and being confirmed by the supervisor.

1. Statistical traffic estimation—the main application of this estimation is to detect alerts, which are regularly repeated to find their repetition pattern. The patterns of occurred alerts are recognized, and the repetition pattern is derived based on the statistical data of each alert and detects the dissimilarity with these patterns in the future [51,52].
2. Causal relation estimation—it estimates causal relationships between alerts, which predicts next alert occurrence and detects attacks. The alert sequence (pattern) is used for detecting false cases. Several works have been introduced to analyze the causal relationships between alerts [50,53,54].
3. Reliability degree combination—the Reliability Degree Combination is responsible for combining reliability with the similar alerts [55,56]. Changing the reliability to alerts is proposed based on equivalent alert repetitions. This combination aims to change the priority or severity of an alert based on the other resources. The presented algorithms require a large amount of labeled previous data for generating the probability models.

### 3.4. Hybrid-Based Approach

The hybrid-based approach tries to take advantage of each of the three correlational approaches. Work [57,58] proposed a hybrid model, which provides alert correlation based on statistics, similarity, and knowledge correlation approaches. Their main goal is to enhance the detection and prediction of alerts and recognize attack scenarios.

### 3.5. Comparison of Alert Correlation Approaches

In this subsection, we provide a comparison of existing approaches, which has been outlined in Table 3. The capability metrics for evaluating alert correlation techniques are defined in the following section.

## 4. Generalized Components in Intrusion Alert Correlation Models

**Figure 2:**
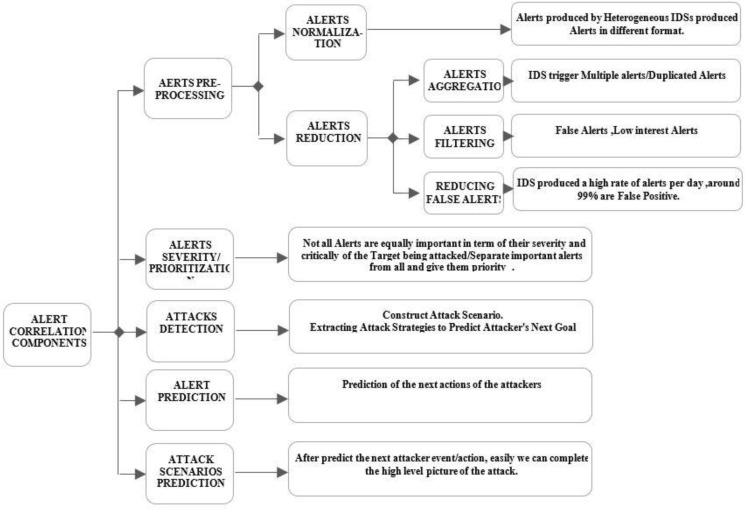
Mapping of alerts correlation components with research problems.

There is much need for Alert Correlation (AC) to ensure the improvement on the quality of alerts that have been made available by NIDS. The prediction of sophisticated attack scenarios requires the development of an effective, efficient, and accurate alert correlation/prediction model. The AC model consists of several tasks that include normalization, reduction, severity/prioritization, attack detection, and prediction to present a viewpoint of network security situations. Generalized components in intrusion alert correlation/prediction models has been shown in Figure 2. The following sub-sections briefly explain each of them.

### 4.1. Alert Normalization/Formatting

Among the preprocessing tasks studied by AC, formatting alerts can be considered as an important initial task. Currently, most organizations implement different types of NIDS (heterogeneous NIDS), so they generate alerts in different data formats. Alert normalization is the process of converting different alert data formats from multiple intrusion sensors into appropriate and acceptable standard formats for other correlation components. The authors of [59] tended to the issue of formatting and standardizing the unformatted alerts. Their work has motivated to design a format called Intrusion Detection Message Exchange Format (IDMEF), which can be received in a wide range of IDS.

**Figure 3:**
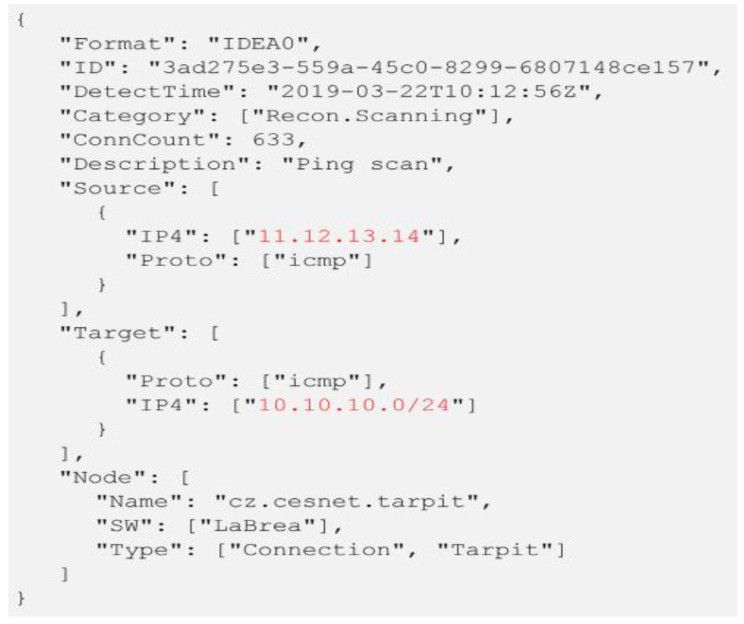
Example of an IDEA alert in JSON format.

Another alert format based on IDMEF is called IDEA (intrusion detection extensible alert) [60]. IDEA uses the latest JSON format instead of XML and adds alert classification. Figure 3 shows an example of alert in IDEA format.

### 4.2. Alert Reduction

NIDS can produce thousands of alerts per day. This flow contains repeated/redundant and low interesting alerts. About 99% of the alerts are false positive [61]. To reduce the alerts, we categorized the related works into two groups: aggregation and filtration.

1. Alert aggregation:

Alerts belonging to the same attack may be generated by the same NIDS or different NIDS. They are recognized by a similar IP addresses source and destination and merged with repeated/redundant alerts. This case increases the dimensionality of created alerts. In fact, redundant alerts are usually false positives.

Aggregation is utilized to group redundant/repeated alerts and represent to as a hyper-alert or single meta-alert. Alerts are clustered (or aggregated) based on attributes/features similarities [59] using predefined rules operator/function. A new cluster is created to represent aggregate clusters, and a new global alert is created to represent the new cluster [62,63]. Clustering is practical because a similarity search between alerts can be performed automatically on many alerts

Another effort attempts to reduce unnecessary alerts by validating alerts with vulnerabilities assessment and then aggregate alerts [64,65]. Kavousi et al. [26] aggregate alerts that have been caused for same attack stage. Zomlot [66] used a support vector matrix (SVM) to reduce alerts, and the non-interested alerts are not removed; they claim it will be helpful to link true attacks. However, reducing redundant alerts does not truly eliminate false positives. Therefore, the next problem is to filter (remove) and verify false positive alerts.

2. Alert filtration—technically, false positives alerts are normally caused by runtime limitations, specificity of detection signature, and environment dependency.

False positives alerts need to be verified and filtered (removed) to ensure effective alert correlation. In [67], a clustering algorithm based on the XML distance measure used to aggregate alerts in clusters is implemented. Each XML file represents a sequence of alerts for a network session. In [68], the authors verify and filter false positive alerts using machine learning, whereas [69] have adopted a fuzzy-based classifier to generate a fuzzy rules classifier to differentiate between alerts as true or false positives using knowledge, while [70] have used genetic based fuzzy classifier to generate the classification rules.

### 4.3. Alert Severity/Prioritization

Not all intrusion alerts generated by NIDS are equally important to the severity and target of the attack [71], so some critical alerts need to be separated and prioritized from the rest of the alert set.

Work by [72] categorized alerts severity into three types: high, medium, and low, the type of severity based on NIDS signature files; while Alsubhi [55] proposed a technique based on fuzzy logic for scoring and prioritizing alerts. Their method evaluates alerts according to several criteria and used fuzzy logic inference mechanism prioritize alerts.

### 4.4. Attack Prediction and Construction

As is known, the prediction of future actions of an attacker is one of the most important goals here. NIDS technologies play a vital role in protecting communication networks from cybercrime. However, these techniques are not very effective in predicting future action of the attacker. Even worse, it generates alerts when attack/intrusion activities occur. A predictive approach is to anticipate and implement potential attacks to prevent damage. Accordingly, the future attack step can be derived after detecting a few steps of the attack in progress. However, anticipating the next actions of attackers is a difficult and important task. Predicting an attack future action can help intrusion prevention systems to function properly. Details about intrusion alert prediction are given in Section 2.

## 5. Intrusion Alerts Datasets

A major challenge for the research community is to obtain suitable datasets for evaluating various research designs in the domains of NIDS [73,74]. A complex and new case of intrusions, new bugs, new security issues, and vulnerabilities are growing every day. The evaluation process of alert correlation research shares the same challenges of NIDS research, as AC is a complementary system to NIDS. One of these challenges is the unavailability of enough datasets. However, a number of datasets have been used for testing Alert Correlation researches, such as KDD 99 [75], DARPA 2000 [76,77], Koyto2006+ [78,79], and Defcon [77], and, recently, alert sharing platform (SABU) [80] datasets. There are verities of security issue concerning datasets for evaluating alert correlation. The authors of [73] listed some issue regarding the use of datasets, which include data privacy, obtaining approval from the data owner, the scope of evaluative datasets, a documentation problem, understanding datasets, data labeling, availability of evaluative datasets, and discrepancies in evaluative datasets.

**Figure 4:**
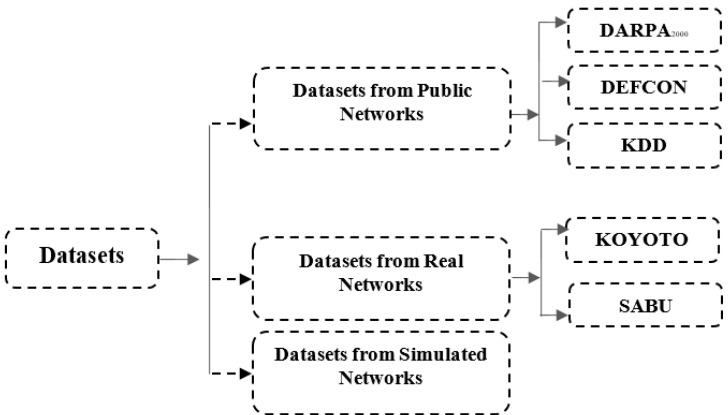
The source of datasets.

1. Data privacy—realistic data are not allowed to be shared among users due to security policies, sensitivities of realistic data, lack of trust, and risk of disclosing digital information.
2. Getting approval from data owner—some data owners require approval to obtain access to the dataset, and this approval process is frequently delayed.
3. The scope of evaluative datasets—most publicly available datasets become out of date and unsuitable for making strong scientific claims because of variability in network segments.
4. Different research objectives—the aims and methods of studies are factors that influence the choice of datasets.
5. Data labeling—some available datasets are manually labeled datasets, while some are packet traces without identifiers, which influences the validity of the datasets.
6. Source of evaluating datasets—there are three conceivable approaches to making more reasonable datasets for assessing the alert correlation researches to limit the effect of challenges. The datasets can be extracted from any of the general population (for example, DARPA), local area systems, and genuine systems (Koyto2006+), as shown in Figure 4.
7. Alert sharing platform (SABU) dataset: This dataset was recently published as the first intrusion alert dataset; it contains intrusion alerts collected from heterogeneous intrusion detection systems in different organizations [80]. Alerts are formatted in the (IDEA) format and classified using the eCSIRT.net taxonomy. The IDEA format, as shown in Figure 4, is a descriptive data model that uses a modern JSON structure. Nearly 12 million alerts have been collected in three organization from different data sources (34 IDS, honeypots, and other data sources). This dataset has been used in significant research to test alert correlation and prediction models [3,30,81,82].
8. DARPA 2000 datasets: The DARPA-2000 dataset is among the notable datasets, which are utilized to assess alert correlation research. The datasets are made by the Lincoln Lab, Massachusetts Institute of Technology (MIT) USA [76]. This dataset comprises two multi-stage scenarios, called LLDDOS 1.0 (Scenario One) and LLDDOS 2.0.2 (Scenario Two). Both attack scenarios contain a progression of attack activities to start distributed denial of service assaults (DDoS). A significant number of investigations have been made into this dataset to test the alerts correlation frameworks [20,26,44,57].
9. KDD: The KDD 99 Dataset is created on basis of the data captured in DARPA’98 IDS evaluation program and used for the evaluation of Alerts Correlation researches [83]. KDD 99 network traffic consists of 41 features and is labeled as either an attack activity or normal activity. The attacks are categories as user to root attack (U2R), denial of service attack (DoS), remote to local attack (R2L), and probing attack.
10. Datasets from Real Networks: The second approach is to deal with more sensible datasets from genuine networks, which are a great source of real events. Such datasets, as a rule, require endorsement from administrators of the networks.
11. KYOTO2006+: The Kyoto 2006+ is datasets that have been used for evaluating Alerts Correlation researches [79]. These datasets were created from real network traffic from Nov 2006 to Aug 2009, without human intervention at the University of Kyoto, Japan. They consist of 24 statistical features where 14 conventional features are extracted based on KDD99 datasets and 10 additional features. These feature assist in investigating the happenings within the network.

Datasets from Simulated Networks: The third approach is to set up local area networks to mimic a few attacks in line. There are several researchers in this research domain that applied this approach [84]. The comparison of different types of attacks and methods is shown in Table 4.

## 6. Discussion and Future Research Direction

Predicting the next activities of the attacker is an imperative and difficult task [13,20,81,82]. Prediction encourages intrusion frameworks to respond appropriately before the network is compromised and gives the chances to overcome the benefits of the attacker. However, existing works on conquering the limitations of NIDSs (in term of delivering a high volume of low-quality alarms) do not manage alert correlation [40] and attacker prediction [24] as a proactive approach. The present expectation methods exhibited on intrusion alerts prediction have constrained capacity to anticipate attack situations that have never been experienced. Various researchers exploring AC have recommended that analyzing intrusion alarms created by NIDS gives a brief and a high-level figure when attack exercises have occurred in a timely manner [19,24,26,38,49,71].

AC is a complex multi-organized change process, and the majority of the existing procedure experiences complex relationship rules definition [26] that constrains the capacities of recognizing new attack situations. The capacities are restricted because of hard-coded area information, which is precisely predefined, depending on the learning of human expert. Prediction in known conditions can be unraveled utilizing information base framework or Bayesian derivation with a foreordained structure. The probabilistic and Markov methodologies will function admirably when the environment is obscure yet exceptionally tedious. At the point when nature is obscure yet stationary with restricted social and question assortment, Bayesian and Markov will take some opportunity to learn. It is significant and reasonable to propose an Intrusion Alerts Correlation Prediction Model, which incorporates an expectation of the assailant’s subsequent stage and the aggressor’s new situation. Intrusion detection alert can be expressed as a relational time series (RTS). The NIDS creates cautions as malicious exercises that arrive in time succession.

## 7. Conclusions

The trends and solutions have been highlighted according to the issues and advantages of intrusion alert prediction. The growing interest in the internet, communication networks, telecommunications alternative, and cybercrimes issues have increased the necessity of robust predictive mechanisms. Advanced and modern attacks are faced that exploit the emerging services, as simple attacks are no longer used. The Network Intrusion Detection Systems (NIDSs) are useful if the detected results are reviewed and analyzed to derive security for the current system. NIDS operations generate a high volume of low-quality alerts. However, 99% of the alerts produced by NIDSs, are a false positive. The study has presented an overview of past and recent works in the field of NIDSs and alert correlation. Several NIDS approaches have been discussed that provide taxonomy to show the types of NIDS. The limitation of NIDSs that motivate the alert correlation research has been summarized. Alert-correlation-related works have also been discussed. Different approaches including similarity-based, knowledge-based, statistical-based, and hybrid-based approaches have also been discussed. The study concludes that the existing intrusion alert prediction systems have limited prediction capabilities.

## Figures and Tables

**Table 1:** Comparison of this survey and existing surveys. (✔: Topic is covered, ☒ the topic is not covered).

Survey (Year)	Alert Correlation Approaches	Alert Prediction Methods	Dataset Issue
Sadoddin (2006) [15]	✔	☒	☒
Mirheidari (2013) [16]	✔	☒	☒
Salah (2013) [14]	✔	✔	☒
Yu Beng (2014) [13]	✔	☒	☒
Proposed Survey	✔	✔	✔

**Table 2:** Summarizes intrusion alert prediction methods.

Methods	Advantages	Disadvantages	References
Deep Learning	Useful in feature learning.	There is a confusion of how to adopt deep learning in alert correlation applications.	[3,30,31]
Bayesian Network	High accuracy for detecting well-known attacks.	Use complex correlation techniques to discover root-case or attack scenario.	[6,26,27]
Relational Time Series	Performing well in alert reduction, clustering, aggregation, and pattern-matching.	Difficult aspects of selecting representation methods when determining a compatible and adequate similarity measure.	[22,25]
Machine Learning and Data Mining	Performed well in static networks, used to represent a well-defined attack scenarios.	Scalability is the big issue.	[5,19,22,23]
Sequence Pattern Mining	These methods can detect known and unknown attack scenarios.	Complex algorithms.	[20,21]
Network Attack Graph	High accuracy for known attacks.	Complex correlation algorithms.	[19,27]
Plan Library	Correlation result is easy to understand. Discovers all attack scenario variants.	Knowledge build by expert, or predefined scenarios. Scalability problem.	[17,18,32]

**Table 3:** A comparison of existing approaches (Capable = ✔, incapable = ☒).

Approach	Pre-Knowledge or Rule	Alert Reduction	Reducing False Alerts	Alert Prioritization	Extract Attack Scenario	Predict Next Alert	Construct and Predict Attack
Similarity-based	✔	✔	✔	☒	✔	☒	☒
Statistical-based	✔	✔	✔	✔	✔	✔	☒
Knowledge-based	✔	☒	☒	✔	✔	☒	☒
Hybrid-based	✔	✔	✔	✔	✔	☒	☒

**Table 4:** Comparison of different types of attacks and methods.

Used Algorithms	Objective(s)	Accuracy (%)	Datasets	Type of Attacks	References
K-means + KNN	IDS	93.55	KDD-Cup’99	DoS, User to Root (U2R), Remote to Local (R2L) and Probing Attack	[85]
SVM + KNN + PSO	IDS	88.44	KDD-Cup 99	DoS, User to Root (U2R), Remote to Local (R2L) and Probing Attack	[86]
HC + SVM	IDS	95.72, 69.8	KDD Cup 1999, 1998 DARPA	DoS, User to Root (U2R), Remote to Local (R2L) and Probing Attack	[87,88]
RF + AODE	IDS	90.51	Kyoto	Various attacks against honeypots	[89]
FL + ES	Network forensics	91.5	DARPA 2000	DDoS, DARPA attacks	[90]
FL + GA	IDS	94.6	DARPA-KDD99	DoS, User to Root (U2R), Remote to Local (R2L) and Probing Attack	[91]

SVM: support vector machine; KNN: K-nearest neighbors; PSO: particle swarm optimization; AODE: average one-dependence estimator; HC: hierarchical clustering; AODE: average one-dependence estimator; RF: random forest; FL: fuzzy logic; ES: expert system; GA: genetic algorithm; IDS: intrusion detection system.
